# Retraction Note: Grammar enhancement in EFL instruction: a reflection on the effects of self-evaluation, teacher support, and L2 grit

**DOI:** 10.1186/s40359-025-02875-x

**Published:** 2025-05-19

**Authors:** Kelu Wang

**Affiliations:** School of Foreign Languages, Leshan Normal University, Leshan, 614000 China

**Retraction Note: BMC Psychology (2024) 12:15**

10.1186/s40359-023-01504-9

The editor and the publisher have retracted this article. The article was submitted to be part of a guest-edited issue. An investigation by the publisher found a number of articles, including this one, with a number of concerns, including but not limited to compromised editorial handling and peer review process, inappropriate or irrelevant references or not being in scope of the journal or guest-edited issue. Based on the investigation’s findings the editor therefore no longer has confidence in the results and conclusions of this article.

Author Kelu Wang disagrees with this retraction.

